# Deficiencies in the transfer and availability of clinical trials evidence: a review of existing systems and standards

**DOI:** 10.1186/1472-6947-12-95

**Published:** 2012-09-04

**Authors:** Gert van Valkenhoef, Tommi Tervonen, Bert de Brock, Hans Hillege

**Affiliations:** 1 Department of Epidemiology, University of Groningen, University Medical Center Groningen, Groningen, The Netherlands; 2 Faculty of Economics and Business, University of Groningen, Groningen, The Netherlands; 3 Econometric Institute, Erasmus University Rotterdam, Rotterdam, The Netherlands

## Abstract

**Background:**

Decisions concerning drug safety and efficacy are generally based on pivotal evidence provided by clinical trials. Unfortunately, finding the relevant clinical trials is difficult and their results are only available in text-based reports. Systematic reviews aim to provide a comprehensive overview of the evidence in a specific area, but may not provide the data required for decision making.

**Methods:**

We review and analyze the existing information systems and standards for aggregate level clinical trials information from the perspective of systematic review and evidence-based decision making.

**Results:**

The technology currently used has major shortcomings, which cause deficiencies in the transfer, traceability and availability of clinical trials information. Specifically, data available to decision makers is insufficiently structured, and consequently the decisions cannot be properly traced back to the underlying evidence. Regulatory submission, trial publication, trial registration, and systematic review produce unstructured datasets that are insufficient for supporting evidence-based decision making.

**Conclusions:**

The current situation is a hindrance to policy decision makers as it prevents fully transparent decision making and the development of more advanced decision support systems. Addressing the identified deficiencies would enable more efficient, informed, and transparent evidence-based medical decision making.

## Background

### Motivation

Health care policy decision makers such as drug regulatory authorities, reimbursement policy makers and guideline committees routinely evaluate the efficacy and safety of medicines, as well as other factors such as costs. Clinical trials provide the pivotal evidence for drug efficacy and safety. The ability to efficiently identify and make use of the results of existing clinical trials is critical to evidence-based policy decision making.

Until recently, journal publications were the only generally available source of trial designs and results. Thus, systematically reviewing the medical literature for the clinical trials that address a specific topic is of central importance to evidence-based health care policy [1,2]. This provides decision makers with a coherent overview of the current evidence, and also helps to set the agenda for future clinical research [3,4]. However, systematic reviewing is currently not feasible for most decision makers, because it is time consuming and expensive.

Therefore, most decision makers will have to rely on published systematic reviews. However, this is problematic because the review may not match the needs of the decision maker. Thus, even when a relevant systematic review is available, there may be a need to go back to the underlying trial data, especially for quantitative decision modeling. It may additionally be necessary to update or extend the review, or to combine several of them. Doing so also requires access to the underlying trial data, but these are not commonly reported. This is a serious limitation to the efficiency of both evidence-based decision making and systematic reviewing.

Thus, the quality of health care policy could be improved if systematic reviews could be performed for whatever decision is currently at hand, ideally even on demand. This would require enormous improvements to the manner in which clinical trials evidence is made available. Although efforts to standardize the information systems for the management and regulatory submission of clinical trials have been successful [5-7], this has not so far resulted in similar improvements in the dissemination of clinical trial evidence. A comprehensive overview of the various information systems that store and process clinical trials information could identify the gaps in information transfer that limit the efficiency of systematic reviews and consequently health care policy decision making.

### Systematic review

The need to identify and summarize the evidence for decision makers is evident from the sheer scale of the available information: PubMed alone indexes nearly 20 million publications from over 5,500 journals, and this is only a selected subset of the biomedical literature [3]. Systematic review addresses this need and consists of three steps: literature screening, data extraction, and reporting. In the first step, literature databases are searched, yielding a set of potentially relevant publications. These are screened for suitability, which results in them being included in, or excluded from, the review. Because literature searches are often inaccurate, thousands of publications may need to be screened. Moreover, to ensure comprehensiveness and avoid bias, multiple databases have to be searched [8] and multiple publications of a single trial have to be identified as such. Once the relevant trials have been identified and the corresponding reports retrieved, the data have to be extracted from the reports. Finally, the collected data are summarized and combined (e.g. using meta-analysis), and reported in a journal article or a technical report. Typically only this final product is made available, even though making the results of the screening step and the extracted data available would greatly enhance the efficiency of future systematic reviews and decision making. Thus, to assess the efficiency of clinical trials results dissemination, systematic review should not be ignored.

The difficulty of performing a systematic review also impacts the quality of systematic reviews themselves: it leads to reviews that focus on a single treatment or a pair of treatments. Consequently, for one particular therapeutic indication many competing reviews may be available, that each provide only a small part of the overall picture [9]. This has led to ‘overviews of reviews’ or ‘umbrella reviews’ summarizing the results of several existing reviews [10]. Umbrella reviews generally merely repeat the pooled summaries of treatment effects from the original reviews, but it has been argued that they may lead to misleading and inconsistent conclusions [9]. An approach based on the individual studies is therefore preferable but labor-intensive if the data are not available in a structured format.

### Scope and objectives

The aim of this paper is to identify opportunities to enhance the efficiency of systematic review and evidence-based decision making, supported by a broad and useful overview of the current state of the art in the transfer and availability of clinical trial evidence. To these ends, we provide a critical overview of existing systems and standards that support the dissemination of clinical trial results.

Because publicly available clinical trial results are nearly always aggregated (at the population level) rather than reported per patient, and because most decision makers base their decisions on such data, we limit the scope of this paper to systems and standards for the aggregate level.

## Methods

We included academic publications and websites of manufacturers or standardization bodies that describe information systems or standards that deal with the transfer and availability of aggregate-level results of clinical trials. We also considered review articles and peer-reviewed position papers related to such information systems.

We identified relevant publications through key word searches using Google, Google Scholar, ISI Web of Science and PubMed (last searched May 2011). We also screened the reference lists of included publications. In addition, through our participation in the Escher project of the Dutch Top Institute Pharma (TI Pharma), we were able to engage in discussions with many experts from the pharmaceutical industry, regulatory authorities, and academia.

Publications (both peer-reviewed articles and web pages published by companies or standardization bodies) were screened for eligibility using titles and abstracts (if applicable). Potentially relevant publications were read in full. If a peer-reviewed article and a web page conveyed (nearly) identical information, only the peer-reviewed work was included. Moreover, web pages were excluded if the source was not considered authoritative for the subject matter. Included publications were summarized using keywords, and especially important sections were highlighted for later reference. For each system and standard we collected the context in which it is used, its purpose, its defining features, the types of data it handles and/or produces, its connection to other systems or standards, and expected future developments.

## Results

**Figure 1:**
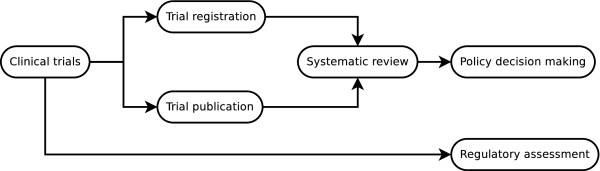
An overview of the processes dealing with clinical trials information and how they relate to each other.

In this section, we present the identified systems grouped according to the processes they support. These are publication in the scientific literature, trial registration, systematic review, and regulatory assessment. Figure 1 shows how these processes relate to each other, to the operation of the trial itself, and to policy decision making. Subsequently, standards and data models relevant to the dissemination of aggregate level results of clinical trials are discussed.

### Scientific literature

Pharmaceutical industry and other investigators may choose to summarize selected results of clinical trials in manuscripts submitted to peer-reviewed scientific journals. A clinical trial may result in any number of publications, from none to dozens. Unfortunately, such publications frequently lack sufficient information to allow the reader to judge whether the trial was rigorously conducted. The CONSORT statement [11,12] aims to improve the situation by providing guidance on the proper reporting of clinical trials. Nevertheless, trial reporting is often still inadequate [13] and selective outcome reporting is common [14].

Reporting clinical trial results in text-based articles rather than properly structured data sets makes computational processing of the results practically impossible [15]. Moreover, although peer review is essential to guarantee the quality of such articles, publication in scientific journals scatters the results throughout the scientific literature. The problem of identifying and accessing clinical trial publications is addressed by abstract databases and search engines, most notably PubMed (http://pubmed.com/), backed by the MEDLINE database, and EMBASE (http://embase.com/), which maintains its own database; see Table 1 for their coverage. Both databases label abstracts using controlled vocabularies, allowing the restriction of searches to clinical trials, controlled clinical trials, or systematic reviews of clinical trials. However, not all abstracts might be labeled, which is why systematic reviewers often broaden their search beyond these categories. PubMed’s Clinical Queries (http://pubmed.com/clinical) provide optimized search strategies that have been empirically validated for this use [16]. It might be useful in these cases to annotate which abstracts *do not* belong to reports of clinical trials. Research is ongoing to improve the accuracy of search results through text processing of abstracts [17] and to aid the screening process by ranking the search results [18]. The Cochrane CENTRAL database of trial publications [19], kept up-to-date by the non-commercial Cochrane Collaboration (http://www.cochrane.org/), provides links to publications known to describe randomized controlled trials. It is quarterly updated from MEDLINE, EMBASE, and databases of specialized Cochrane groups [20] Records that are identified as clinical trials are reported back to MEDLINE.

**Table 1:** Coverage of abstract databases PubMed and EMBASE, and the Cochrane Library

	**all records**	**since 2000**	**source**
**PubMed/MEDLINE (1 Jan 2011)**			
PubMed records	19,569,568	6,628,156	(1)
Identified clinical trial	457,378	171,025	(1)
Identified randomized controlled trial	293,963	156,496	(1)
Identified meta-analysis	25,723	21,146	(1)
Indexed journals	5,543	1,287	(2) 17 May 2011
**EMBASE** (17 May 2011)			
EMBASE records	∼ 24 M		(3)
EMBASE journals	≥ 7,500		(3)
**Cochrane library** (17 May 2011)			
Clinical trials (CENTRAL)	645,086	286,418	(4) issue 2 of 4, Apr 2011
Cochrane reviews	4,621	4,621	(4) issue 5 of 12, May 2011
Other reviews	14,602	12,683	(4) issue 2 of 4, Apr 2011
All reviews (Cochrane + Other)	19,223	17,304	
Cochrane protocols	2,020	2,020	(4) issue 5 of 12, May 2011
**sources**			
1. http://www.nlm.nih.gov/bsd/licensee/baselinestats.html			
2. http://www.nlm.nih.gov/tsd/serials/lji.html			
3. http://embase.com/info/what-is-embase/coverage			
4. http://thecochranelibrary.com/			

When clinical trial results were only published in scientific journals, the decision whether to publish the results was completely left to the investigator, which led to incomplete trial reporting and publication bias [21]. For example, over half of the clinical trials that supported successful new drug applications made to the FDA had still not been published 5 years after the medicines’ market approval [22]. This is a serious problem that can lead to incorrect conclusions from a systematic review.

### Trial registration

As early as 1986 the registration of trials in advance was proposed as a solution to publication bias [23]. The *trial bank* concept proposes to take the registration of trials even further by recording not only the existence of a trial, but also the study protocol (in advance) and the results (after completion) in a “machine readable” way [15,24]. For trial banks to be successful, all trials must be entered in a way that conforms to a single machine-readable data model [24,25]. The Global Trial Bank project was set up in 2005 to create a practically usable trial bank [26], but in 2008 it was put on hold due to lack of funding [27].

The FDA Modernization Act of 1997 made the US the first country to make trial registration a legal requirement. To implement this legislation, the ClinicalTrials.gov registry was launched in February 2000 [28]. The initial installment focused on providing a record of trials for enabling patient recruitment and investigator accountability. In 2004, both the World Health Organization (WHO) and the International Committee of Medical Journal Editors (ICMJE) released statements in support of the prospective registration of clinical trials. This policy has been widely adopted and now assures that the existence of most recent trials is known [29]. Subsequently, various organizations, including the WHO, have called for a full disclosure of the trial protocol (including amendments) and results [30-36]. In the US, recent legislation [37] has required protocol registration since December 2007, basic results reporting since September 2008, and Adverse Drug Events (ADEs) reporting since September 2009 [38]. Other governments with policies requiring prospective registration include the EU, India, Argentina, Brasil, Israel and South Africa [39].

To register a trial in ClinicalTrials.gov [40], researchers enter summary protocol information [41] when their studies are initiated, and subsequently create the results section [42] when the data collection for at least one primary outcome measure is complete. The ClinicalTrials.gov staff will review the results data after their submission. The data are reported in a structured tabular format and some meta-data, such as units of measurement or the use of standard vocabularies, can also be provided. Limited support for reporting statistical analyses is offered; these analyses are tied to specific results tables. Study protocols have long been available in XML format, and the retrieval of results in XML format was added in December 2011 [43].

Other countries have set up their own registries. Since 2004, the European Medicines Agency (EMA) has established clinical trial registration in accordance with the EU Directive 2001/20/EC through the EudraCT system. EudraCT was opened to the public only recently as the EU Clinical Trials Register, on 22 March 2011 [44], and the records are being released in a staggered fashion. Currently 17,102 [45] of over 28,150 registered trials [46] are available. Another international registry is the Current Controlled Trials Ltd.’s ISRCTN registry, which has been in operation since 1998 [47]. It provides a semi-structured textual representation of the trial protocol, but no results. A number of countries have open national registries that generally record information in a way similar to ISRCTN. All of these registries are less sophisticated than ClinicalTrials.gov.

In order to unify trial registration world-wide, the WHO International Clinical Trials Registry Platform (ICTRP) was established following the Ministerial Summit on Health Research in November 2004. The goal of the ICTRP is to create “a network of international clinical trial registries to ensure a single point of access and the unambiguous identification of trials” [48]. This network of trial registries, the WHO Registry Network, was formally launched in 2007. In March 2012, 14 primary registries were listed on the ICTRP website (see Table 2). The ICTRP also provides a search portal that collects and indexes some basic information on trials from most of the primary registries and attempts to group trials that are registered in more than one registry in the search results. The search portal provides a textual description of the trial design as well as a link to the primary registry. Table 2 gives an overview of the WHO primary registries and ClinicalTrials.gov, with the number of included studies and whether or not they are indexed by the search portal. ClinicalTrials.gov is by far the largest registry, containing more than 7 times the number of trials available in the second largest registry (EU Clinical Trials Register), and 69% of all trials in the registries (not taking duplicates into account). Moreover, ClinicalTrials.gov is the only registry that registers results. In January 2010 it had published the results of 1,156 studies, which had increased to 5,436 studies by March 2012.

**Table 2:** ClinicalTrials.gov and the 14 WHO primary registries, with the number of registered trials per 19 March 2012 (‘studies’), whether the register is indexed by the WHO search portal (‘indexed’) and whether the registry also enables results publication (‘results’)

**Register**	**Studies**	**Indexed**	**Results**
ClinicalTrials.gov (United States)	122,758	yes	yes (5,436)
*European Union* Clinical Trials Register	17,102	no	no
ISRCTN register (international)	10,465	yes	no
*Japan* Primary Registries Network	8,329	yes	no
*Australian New Zealand* Clinical Trials Registry	6,369	yes	no
*The Netherlands* National Trial Register	3,187	yes	no
Clinical Trials Registry - *India*	2,499	yes	no
*Iranian* Registry of Clinical Trials	2,449	yes	no
*Chinese* Clinical Trial Register	2,004	yes	no
*German* Clinical Trials Register	831	yes	no
*Cuban* Public Registry of Clinical Trials	392	yes	no
*South Korea* Clinical Research Information Service	379	yes	no
*Brazilian* Clinical Trials Registry	131	no	no
*Pan African* Clinical Trial Registry	97	yes	no
*Sri Lanka* Clinical Trials Registry	71	yes	no

Although a decentralized system of federated registries (both national and multi-national) seems cumbersome and may cause duplicate registration, there are important reasons why this method is to be preferred to a centralized approach [49]: national registries, for example, are in the position to ensure complete registration in their region of influence and are perfectly aligned with the local political situation. As long as the different registries are sufficiently interoperable, an overarching organization such as the ICTRP can aggregate their databases.

The increased transparency enabled by trial registration offers new opportunities for evidence-based medicine and will likely lead to an increase in the number of systematic reviews that are undertaken [50]. However, the current registries contain only text-based or semi-structured information and lack a common coding system, for example for labeling interventions. The amount of protocol information registered is often insufficient to judge the validity of reported results and the problem of identifying all relevant studies has not yet been solved [29]. In addition, the publicly available information may be incomplete or even “largely incomprehensible” [38]. The call for federated, open access, mandatory results databases continues [32-36], and it is likely that the trend toward open and complete registration of results will continue.

### Systematic review

The process of systematic review produces data and meta-data that is potentially useful for future reviewers and decision makers (see Background). The following assesses whether the existing information systems enable the dissemination of this information.

The Cochrane Collaboration provides several databases to support reviewers. Besides the CENTRAL database of clinical trials (see Section Scientific literature) the most relevant ones are the Cochrane Database of Systematic Reviews, in which Cochrane reviews are published [51], and the DARE database of other reviews. Table 1 provides statistics regarding the scope of the library. In contrast to the traditional journal publications of systematic reviews, which usually provide data in tables or figures, the Cochrane Reviews incorporate descriptions and results of the original studies. However, the published dataset has many data elements removed and the use of the data is restricted by license.

There are several software programs to aid in systematic review and meta-analysis, such as Comprehensive Meta-Analysis, MetaWin, MetaStat and MetaAnalyst. Moreover, many general-purpose statistical programs, such as SPSS Statistics, SAS Statistics, Stata, and R, have meta-analysis functionality. In general, dedicated meta-analysis software will provide easier data entry and management, while statistical programs will offer more powerful tools for analysis. The Cochrane Collaboration also provides the Review Manager software for performing systematic reviews (http://ims.cochrane.org/revman). Review Manager is unique in that it provides not only data analysis and management features, but includes functionality to write the full systematic review report. Indeed, the Review Manager file itself is submitted to the Cochrane library for review and eventual publication. Unfortunately, all of these systems lack sufficient meta-data to enable automated processing.

Finally, published systematic reviews are usually presented in a textual format without the underlying dataset, making it difficult to perform additional analyses that may be required for decision making. Thus, the inclusion of data on a new trial or new compound, as would be required for regulatory decision making, is also impossible. In conclusion, systematic review currently represents a missed opportunity to introduce additional structure to the available clinical trials information.

### Regulatory assessment

After a pharmaceutical company develops a drug, it compiles the evidence collected from the discovery and development processes into a dossier that is submitted to the regulators who decide upon its market authorization. Submissions to the EMA and the national medicines boards in Europe are mainly text-based, containing aggregate-level results of clinical trials based on the applicant’s statistical analysis. The Food and Drug Administration (FDA) requests the submission of patient-level data, which is unlikely to become publicly available and as such is out of scope for this paper. The dossier forms the basis on which regulators assess the benefit-risk profile of a new drug. Although the clinical trial results are pivotal in this assessment, the decision is only indirectly based on them, as the decision making process is based on informal discussion between experts. While the decision may still be of high quality, this informal framework does not allow pharmaceutical companies or patients to discern how different pieces of the evidence weigh in on it.

The EMA publishes the European Public Assessment Reports (EPARs) of all centrally approved or refused medicines on its website. Note that this does not include all applications submitted to the EMA, as they can be withdrawn before a decision is reached [52]. The EPAR contains information on all trials, but is completely textual without a semantic structure. Moreover, its information is directly derived from the submission by the applicant, while there is no standardization concerning what information should be provided, or in which format. Trials submitted to the EMA are required to be registered in EudraCT and will thus also be made known to the public through the EU Clinical Trials Register.

### Standards and data models

A common standard of how clinical trials are performed and how their results are presented would make the process of systematic review more reliable and less laborous [15]. Some progress has been made by ClinicalTrials.gov, which currently registers and displays aggregate results. To do so, ClinicalTrials.gov has developed their own model, the Data Element Definitions (DED) [41,42]. This model allows the reporting of aggregated outcome data and statistical analyses to some extent, but the information cannot be processed automatically because most fields are free text. This also means that finding all trials that are relevant to a specific patient condition is inaccurate, and thus requires overly broad search terms [53]. This lack of standardization and interoperability among registries and other databases should be addressed in the near future.

Several projects aim to enable general purpose re-use of clinical trials information, e.g. for cross-study analyses. We identified three such projects. The first is the Biomedical Research Integrated Domain Group (BRIDG) project, a collaboration between the Clinical Data Interchange Standards Consortium (CDISC), Health Level 7 (HL7), the National Cancer Institute (NCI) and the FDA that aims to bring together the common elements of their various standards into a complete data model for clinical trials [54]. The BRIDG model is implementation-independent in the sense that it specifies the problem domain, not a specific solution. For example, unlike some other CDISC standards it does not specify the format in which to submit data to the FDA. BRIDG is subdivided between the protocol representation, study conduct, adverse event and regulatory perspectives. Unfortunately, a data analysis perspective is currently missing as there is no adequate standard for the modelling of statistical analyses. In short, the BRIDG model is accurate as regards the management of a single clinical trial, but not as regards cross-study analysis [55]. For example, the study population and eligibility criteria, outcomes and the measures used to assess outcomes do not have a sufficiently deep semantic structure.

To enable cross-study analyses and efficiently finding relevant trials, the Human Studies Database (HSDB) project aims to share fully machine understandable representations of study design information between institutions [55]. HSDB is developing the Ontology of Clinical Research (OCRe), which defines the concepts that should be accessible across the individual institutions’ databases. At the time of writing, OCRe included a study design representation derived from BRIDG [55], a study design typology [56], the ERGO formal machine readable representation of eligibility criteria [53], and a model of study outcomes that separates the phenomena of interest from the variables that encode them [55]. While OCRe is a promising effort, its representation of study design is far from comprehensive, and it completely lacks a model for trial results.

Finally, the Ontology Based eXtensible conceptual model (OBX) is another ontology for representing clinical trials [57,58]. Its aim is to make the results of immunology studies available for data re-use and re-analysis. The OBX also incorporates study design representation ideas from BRIDG and the ClinicalTrials.gov DED [57]. While it appears successful in developing a broadly applicable data model for biomedical studies, and also allows the inclusion of trial results, it would appear that OBX suffers from similar shortcomings as regards the depth of modelling as BRIDG does.

All of the discussed models rely on an external coding system for their clinical content. Such coding systems, known as controlled terminologies of clinical terms, are an important first step in the application of information technology to medicine [59]. There are many controlled terminologies for medicine, often developed for specific applications, but unfortunately there is as yet no standardization of which ones should be used, and there is no accurate mapping between them [60,61]. For example, in clinical research the Medical Dictionary for Regulatory Activities (MedDRA) is used to code ADEs, while the healthcare area prefers the Systematized Nomenclature of Medicine, Clinical Terms (SNOMED CT) dictionary. This hinders the interoperability of the various information systems being used.

## Discussion

Having reviewed the information systems and standards dealing with the information from clinical trials, we will now summarize their deficiencies concerning the integration of clinical trials information from different resources, discuss how the status quo could be improved and identify directions for future research.

### Identified deficiencies

Systems and standards oriented towards the management of single studies are relatively mature, but this is not the case for cross-study analysis. There are no known large, successful, and publicly available data warehouses, nor any standards that would enable cross-study analyses of aggregate level results. Considerable effort is required to harmonize the current clinical research standards. Important areas that require standardization are the representation of statistical analyses and aggregate results, as well as complex semantic structures such as patient eligibility criteria. None of the general purpose data models being developed are yet in widespread use, and from the perspective of capturing the designs and results of clinical trials in a reusable way, none of them are close to completion.

Although much effort is spent to publish the results of clinical trials, the current systems do not facilitate optimal use of the information. The journals and abstract databases that publish the trial results do not preserve the results’ structure and thus require manual data extraction. Moreover, relevant articles are hard to identify and the retrieval of all available studies cannot be guaranteed. Public registries are meant to improve the efficiency and reliability of the identification of relevant studies, but the available data is not sufficiently structured to realize this. Moreover, the systems that currently deal with clinical trials results are not interlinked nor do they use interoperable standards. In short, there is not yet a comprehensive system of structured machine-understandable databases that contains descriptions of the design, execution, and summary-level results of individual trials. This situation hinders systematic review and makes cross-study analyses and data-mining prohibitively difficult. Thus, current infrastructure is focused on text-based reports of single studies, whereas efficient evidence-based medicine requires the automated integration of multiple clinical trials from different information resources.

Moreover, while systematic review collects and appraises the available evidence that is relevant to a certain question, the results are published in an unstructured format. This makes it hard to use the underlying data to inform evidence-based decisions, to verify the analyses, to update the review or to perform a combined analysis of several reviews for an umbrella review. The effort spent on literature screening and data extraction does not result in availability of this information for future reviewers, leading to duplication of effort.

**Figure 2:**
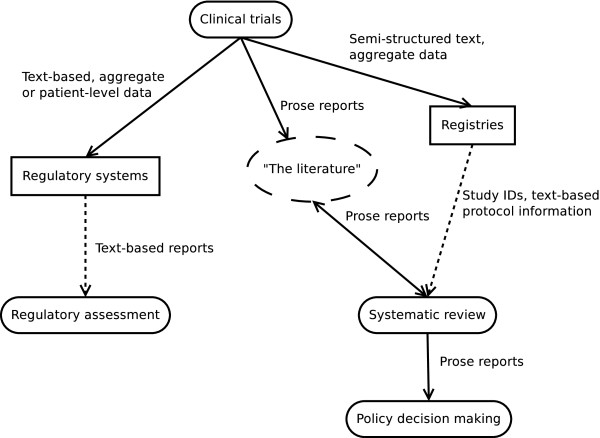
In the current system of clinical trials results dissemination, data are collected in three separate systems (not including the organization that performs the trial).

Therefore, the current systems are unnecessarily burdensome and do not sufficiently facilitate reuse of the information. Figure 2 visualizes the current results dissemination process. Due to these shortcomings, decisions are not explicitly linked to the underlying evidence, leading to a lack of transparency, traceability, and reproducibility that is harmful for all stakeholders.

Importantly, the perceived lack of transparency in regulatory decision making may erode public trust in drug regulation and the pharmaceutical industry. More explicit quantitative decision models would enable a more transparent and reproducible regulatory process, as well as a clearer communication of the requirements to the industry. However, for most real-world decisions it is currently too expensive to include all the evidence. This difficulty of accessing existing data is not only relevant to regulators and the industry, but also to reimbursement organizations, prescribing physicians and patients.

### Proposed future situation

Now, we consider how these deficiencies might be addressed. Let us assume for a moment that a comprehensive machine-understandable standard were available for the design and aggregate level results of clinical trials. Then, it would be better for those submitting the data if both regulators and registries used this format, rather than a number of disparate formats. In addition, journal publications could easily be supplemented with data in this format.

Availability of a standard alone, however, is not enough to enable efficient access to the evidence. The data sets also need to be collected and made available in such a way that relevant clinical trials are easily identified. For this a collaborative (federated) system of databases should be established to capture all clinical trials data. Some of the stakeholders (e.g. regulators and registries) may require that data be submitted to a database that they control so that they can ensure the integrity of the data. This is fine as long as (1) the databases are interoperable and enable access to the information in the same format, (2) there is a single point of access through which the different databases can be identified and located, and (3) duplicate entries can be easily identified. It seems more likely for such a combination of databases to emerge from the current registries than a single centralized system.

**Figure 3:**
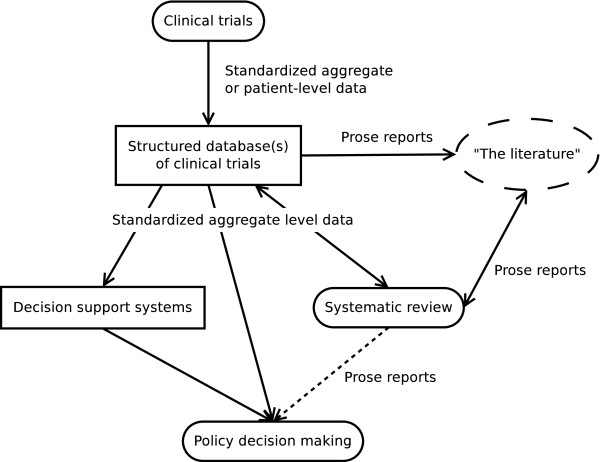
**The alternative solution for clinical trials results dissemination proposed in this paper: harmonization of the different systems to create a unified platform for evidence-based decision making.** Regulatory assessment has been merged into general policy decision making.

A comprehensive record of clinical trials in a machine-understandable format would make systematic review and consequently evidence-based decision making much more efficient. Decisions could then finally be explicitly linked to the underlying data (traceability). In addition, this could also enable a new generation of decision support systems for health care policy decision makers. The proposed future situation is visualized in Figure 3.

However, a suitable general-purpose data model is not likely to become available in the near future. Further, the usefulness of any data model should be demonstrated to the industry and other stakeholders before putting it into practice. We argue that the requirements for a general purpose data model for cross-study analysis and decision making are not sufficiently well known at the moment. Therefore, analysis tools and decision support systems that ensure data extraction done for the analysis can be shared with other researchers should be developed first, to illuminate these requirements.

### Research directions

In order to attain the desired future system for aggregate-level clinical trial results dissemination, we identify concrete research directions for medical informatics, decision making and statistics researchers. Progress on each of these topics can be made in parallel.

• policy decision making based on clinical trials – to enable a direct and explicit link between the decision and the supporting evidence in drug regulation, reimbursement policy and guideline formulation

• Development of a platform to share structured systematic review data sets

• Discovery or creation of incentives for systematic reviewers to share the results of literature screening and data extraction

• Identification of the core data elements and modeling that are needed to increase the accuracy of literature searches

• Automated tagging and data extraction to facilitate transition to more structured data sets

• Development of search tools to integrate querying of abstract databases and registries

• Development of methods to identify duplicate trial publications and registrations

• Development of a comprehensive data model for clinical trials and their aggregate level results

### Limitations

As with any review paper, there is a risk that relevant publications have not been identified, either because the search terms were not broad enough, or because relevant studies were not identified as such based on their title and abstract. We acknowledge that the broad scope of this particular review increases that risk.

The nature of the collected information necessitates a qualitative synthesis, and the identified deficiencies are at least partially subjective. The future that we propose is based on the premise that a standard for aggregate clinical trial data will become available. Unfortunately, it is unclear how and when this could be realized. Finally, the list of proposed research directions is sure to be incomplete, and we hope the present paper will ignite discussions on this topic.

## Conclusions

We reviewed the existing systems and standards dealing with aggregate level results of clinical trials. The transfer of evidence to scientific journals, public registries, and regulators is a largely ad hoc and text-based affair. In part, this is because there are currently no data standards that enable cross-study analyses. We have argued that the lack of a standardized, federated system for results dissemination leads to gaps in the transfer and availability of evidence to the relevant decision makers. As long as such a system does not exist, systematic review will remain an incredibly inefficient ad-hoc process, and evidence-based decision making will remain unnecessarily difficult. We believe that these difficulties lead to a lack of transparency in health care policy decision making, which threatens public trust in the decision makers.

In the future, results registries and regulatory systems should be harmonized and federated to create a system of databases that forms the core of a more automated and efficient process of systematic review and evidence-based decision making. In addition, systematic reviews are currently a missed opportunity to introduce additional structure to the domain of clinical trials information, which should be addressed by more complete dissemination of their results. Although this vision is still far from realized, current trends seem to support this direction. Future work should not only focus on developing the ‘ideal’ data model for all of clinical research (justly called a monumental task) but start by creating useful tools to support decision makers and systematic reviewers. Availability of such tools will lead to increased demand for an accessible evidence base and to a better understanding of its requirements.

## Competing interests

The authors declare that they have no competing interests.

## Authors’ contributions

GvV carried out literature and internet searches and drafted the manuscript. TT carried out additional searches and drafted parts of the manuscript. BdB drafted parts of the manuscript. HH coordinated the study and helped to draft the manuscript. TT, BdB and HH critically commented on many versions of the manuscript. All authors read and approved the final manuscript.

## Authors’ information

GvV is a PhD student for the Escher project of Top Institute Pharma, working on evidence-based decision support for medicines regulation. He has an MSc in Artificial Intelligence.

TT is an assistant professor of Business Intelligence Systems at the Econometric Institute of the Erasmus University Rotterdam. Formerly, he was a post-doc researcher for the Escher project. He has a double PhD degree in Computer Science (University of Turku, Finland) and Management Science (University of Coimbra, Portugal). His main expertise is in Multi-Criteria Decision Analysis (MCDA).

BdB is a professor of Business Information Modelling at the University of Groningen. He is interested in databases and information modelling with interdisciplinary applications to bioinformatics and medicine.

HH is a professor of Cardiology at the University Medical Center Groningen. There, he is also director of the Trial Coordination Center and head of the Data-Management Project. Moreover, he is a clinical assessor for the Dutch Medicines Evaluation Board and clinical expert for the European Medicines Agency (EMA).

## Pre-publication history

The pre-publication history for this paper can be accessed here:

http://www.biomedcentral.com/1472-6947/12/95/prepub
